# EOR-1 and EOR-2 act independently of RAS and WNT signaling pathways in RMED/V neuron specification

**DOI:** 10.17912/micropub.biology.000140

**Published:** 2019-07-31

**Authors:** Xun Huang, Yishi Jin

**Affiliations:** 1 MCD biology, University of California, Santa Cruz, CA95064; 2 Institute of Genetics and Developmental Biology, Chinese Academy of Sciences, Beijing, 100101, China; 3 Neurobiology Section, Division of Biological Sciences, University of California, San Diego, CA92093

**Table 1:**
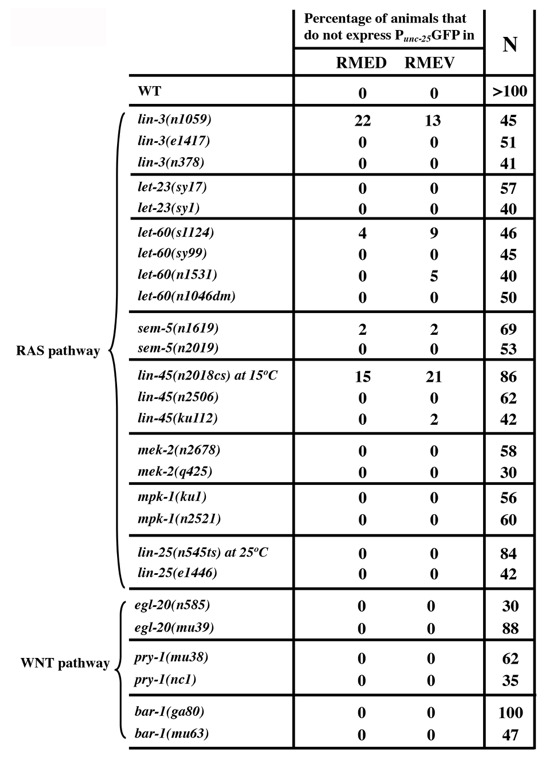
RAS-ERK pathway and the canonical WNT signaling are likely not involved in RMED/V cell specification.P*_unc-25_*GFP expression in RMED/V cells in mutations in RAS or WNT signaling pathway components.

## Description

We found that loss of either *eor-1* or *eor-2* function results in identical differentiation defects in RMED/V neurons (Huang and Jin, 2019a; Huang and Jin, 2019b). EOR-1 and EOR-2 are thought to positively regulate RAS and WNT signaling pathways in vulval cell induction and in P12 cell fate specification (Howard and Sundaram, 2002). Genetic double mutant analysis suggests that *eor-1* and *eor-2* function redundantly with the Mediator complex proteins *sur-2* and *lin-25* (Howard and Sundaram, 2002). We wished to test whether RAS and WNT signaling pathways are involved in RMED/V differentiation. We examined P*_unc-25_*GFP expression in several RAS and WNT mutants (Huang et al., 2004). In the canonical RAS signaling pathway, the EGF-like growth factor LIN-3 binds its receptor LET-23, which then activates LET-60/ras and the MAP kinase cascade that includes LIN-45/raf (MAPKKK),MEK-2/MEK (MAPKK) and MPK-1/ERK (MAPK). We examined strong loss-of-function or putative null mutations in these genes. We detected mild defects in RMED/V cells in *lin-3(n1059)* and *lin-45(n2018cs)* mutant animals. Twenty-two percentage of *lin-3(n1059)* mutants lost P*_unc-25_*GFP expression in RMED, and 13% lost the expression in RMEV (N=45). Fifteen percentage and 21% of *lin-45(n2018cs)* animals at non-permissive temperature did not express P*_unc-25_*GFP in RMED and RMEV, respectively (N=86) (Table 1). However, similar phenotypes were not found in several other alleles of *lin-3* and *lin-45* (Table 1)*.* In addition, mutations in LET-23/EGFR, SEM-5, an adaptor protein, MEK-2/MAPKK and MPK-1/MAPK, had little or no effects on P*_unc-25_*GFP expression in RMED/V (Table 1). The *let-60(n1046)* dominant mutation also did not affect RMEs. *lin-25* has been shown to act in parallel to *eor-1* and *eor-2* in vulva induction, and also did not show any effects on RME. We observed similar results in mutants for the canonical WNT signaling genes including *egl-20/WNT*, *pry-1/Axin* and *bar-1/b-catenin* (Table 1). Therefore, these data suggest that the function of EOR-1 and EOR-2 in RMED/V neurons is likely independent of canonical RAS and WNT pathways.

## Reagents

The mutations used are listed below: Linkage group LGI: *mek-2(n2678)*, *mek-2(q425)*; LGII: *let-23(sy17)*, *let-23(sy1)*; LGIII: *mpk-1(n2521)*, *mpk-1(ku1)*; LGIV: *lin-3(n1059)*, *lin-3(e1417)*, *lin-3(n378)*, *eor-1(cs28)*, *eor-1(ju198)*, *lin-45(n2018)*, *lin-45(n2506)*, *lin-45(ku112)*, *let-60(s1124)*, *let-60(sy99)*, *let-60(n1531)*, *let-60(n1046)*, *egl-20(n585)*, *egl-20(mu39)*; LGV: *lin-25(n545)*, *lin-25(e1446)*, *pry-1(mu38)*, *pry-1(nc1)*, *daf-21(nr2081)*, *daf-21(p673)*; LGX: *sem-5(n1619)*, *sem-5(n2019)*, *eor-2(cs42)*, *eor-2(ju190)*, *bar-1(ga80)*, *bar-1(mu63)*.

## References

[R1] Huang Xun, Jin Yishi (2019). New mutants defective in RMED/V neuron specification are alleles of EOR-1 and EOR-2. microPublication Biology.

[R2] Huang Xun, Jin Yishi (2019). EOR-1 and EOR-2 function in RMED/V neuron specification. microPublication Biology.

[R3] Huang X, Powell-Coffman JA, Jin Y (2004). The AHR-1 aryl hydrocarbon receptor and its co-factor the AHA-1 aryl hydrocarbon receptor nuclear translocator specify GABAergic neuron cell fate in C. elegans.. Development.

[R4] Howard RM, Sundaram MV (2002). C. elegans EOR-1/PLZF and EOR-2 positively regulate Ras and Wnt signaling and function redundantly with LIN-25 and the SUR-2 Mediator component.. Genes Dev.

